# Primum Non Nocere: is shared decision-making the answer?

**DOI:** 10.1186/s13741-016-0042-3

**Published:** 2016-06-16

**Authors:** Ramai Santhirapala, Ramani Moonesinghe

**Affiliations:** Department of Anaesthesia, Royal Surrey County Hospital, Egerton Road, Guildford, Surrey GU2 7XX UK; Division of Surgery and Interventional Science, 4th Floor, University College London, 21 University Street, London, WC1E 6AU UK; Academy of Medical Royal Colleges, 10 Dallington Street, London, EC1V 0DB UK; UCLH NIHR Surgical Outcomes Research Centre, Department of Anaesthetics, 3rd floor, Maples Link Corridor, University College Hospital, 235 Euston Road, London, NW1 2BU UK; Centre for Anaesthesia, University College London, 3rd floor, Maples Link Corridor, University College Hospital, 235 Euston Road, London, NW1 2BU UK; National Institute for Academic Anaesthesia’s Health Services Research Centre, Royal College of Anaesthetists, Churchill House, 35 Red Lion Square, WC1R 4SG London, UK; University College Hospital NIHR Biomedical Research Centre, University College London Hospitals NHS Foundation Trust, London, UK

**Keywords:** Shared decision-making, Patient-centred care, Improvement Science, Education

## Abstract

Surgical ambition is rising, with the Royal College of Surgeons reporting an increase in the number of procedures by a million over the past decade (Royal College of Surgeons. Surgery and the NHS in Numbers. Available from https://www.rcseng.ac.uk). Underpinning, this is a rapidly growing population, especially those in the over 85 age group, coupled with rising perioperative expertise; options for surgery are now present where conditions were once managed conservatively. Matching the right patient to the right procedure has never been so pertinent (Bader, Am Soc Anesthesiol 78(6), 2014).

At the heart of these increasingly complex decisions, which may prove fatal or result in serious morbidity, lies the aspiration of shared decision-making (SDM) (Glance et al., N Engl J Med 370:1379–81, 2014). Shared decision-making is a patient-centred approach taking into account the beliefs, preferences and views of the patient as an expert in what is right for them, supported by clinicians who are the experts in diagnostics and valid therapeutic options (Coulter and Collins, Making shared decision-making a reality: no decision about me, without me, 2011). It has been described as the pinnacle of patient-centred care (Barry et al., N Engl J Med 366:780–1, 2012).

In this commentary, we explore further the concept of shared decision-making, supported by a recent article which highlights critical deficits in current perioperative practice (Ankuda et al., Patient Educ Couns 94(3):328–33, 2014). This article was chosen for the purposes of this commentary as it is a large study across several surgical specialties investigating preoperative shared decision-making, and to our knowledge, the only of this kind.

## Main text

### Background

Shared decision-making (SDM) is the process of using the best available evidence to support patients in making healthcare decisions based on their own values, preferences and beliefs. It is a patient-centred approach which moves away from traditional paternalism, recognising both clinicians and patients as partners in healthcare decisions. The concept of shared decision-making (SDM) was first raised over 30 years ago in the President’s Commission for Study of Ethical Problems in Medicine and Biomedical and Behavioural Research Report (1982), which questioned the validity of informed consent where patients have not had the opportunity to review feasible options and express their preferences. More recently, SDM has been endorsed by a number of national and international bodies, notably the Department of Health, NHS England, King’s Fund and Health Foundation (Coulter and Collins 2011; Health Foundation 2012; Department of Health, Equality and Excellence 2010; Department of Health, Liberating the NHS: greater choice and control 2010, Department of Health, Liberating the NHS: no decision about me without me 2010). With rising surgical ambition, high quality perioperative decision-making is crucial to ensuring patients receive “the care they need and no less, and the care they want and no more" (Coulter and Collins 2011). Taking the example of a patient presenting with knee osteoarthritis, the options available may be physiotherapy, analgesics, arthroscopy, a unicompartmental or total knee replacement. A further example is the patient who presents with metastatic intestinal malignancy where options may range from no further medical intervention, palliation and symptom relief, adjuvant therapies through to defunctioning or curative surgical options which maybe laparoscopic or open. In short, the perioperative pathway is fraught with decisions and the person best placed to make them is the patient, supported by clinicians.

An important step in shared decision-making is ensuring the patient is fully informed of notable risks, benefits and options. Furthermore, patients should have the opportunity to ask further questions with the aspiration that the shared decision is consistent with their lifestyle goals. The preoperative assessment clinic offers a timely opportunity to ensure consent is indeed informed. In addition, this window provides an opportunity to discuss end of life wishes, establishing an advance care plan (ACP) while capacity is intact (Blackwood et al. 2015).

The evidence base for shared decision-making within perioperative care is emerging, and as such, it is challenging to make firm conclusions. Outside the perioperative setting, shared decisions have been demonstrated to improve patients’ self confidence, engagement in healthcare and ultimately satisfaction (Health Foundation 2012). Interestingly, some studies suggest patients may opt for more conservative therapies where the decision is shared (Lee and Emanuel 2013). In the current climate of financial restraint and limited resources, a secondary effect of high quality SDM may be a reduction in inappropriate variation in surgeries. Taking the example of paediatric tonsillectomies in the UK where as great as a sevenfold variation exists in rates between geographic locations, unexplained by severity of illness and resource availability alone (Suleman et al. 2010). Warranted variation based on differing patient preferences should be encouraged; however, unwarranted variation, based for example on professional uncertainty of the benefit of the procedure, should be minimised especially where it leads to inappropriate procedures and potential harm (Appleby et al. 2011). Applying shared decision-making to even minor procedures, which are low in personal stakes for the individual patient but large in volume from a service perspective, could potentially yield immediate cost savings. Whilst supportive evidence of improved patient-reported outcomes and health economics in the perioperative setting will undoubtedly guide specific interventions in the process of SDM, the ethical principle of patient autonomy alone supports the implementation of SDM.

Quality metrics in shared decision-making are difficult to extrapolate to the perioperative setting. Using an adaptation of the Donabedian model, which evaluates healthcare services according to structure, outcomes and processes, Ankuda et al. describe a conceptual model for measuring the quality of preoperative decision-making and report the critical deficits in a unique and innovative study (Ankuda et al. 2014). The aim of this study, which forms the basis of our commentary, was to identify patterns and predictors of deficiencies in shared decision-making and informed consent.

**Fig. 1:**
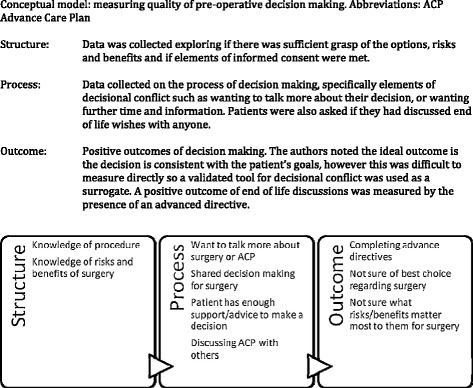
Reproduced from Ankuda et al. 2014. Conceptual model: measuring the quality of preoperative decision-making. *ACP* advance care plan. *Structure*: Data was collected exploring if there was sufficient grasp of the options, risks and benefits and if elements of informed consent were met. *Process*: Data collected on the process of decision-making, specifically elements of decisional conflict such as wanting to talk more about their decision, or wanting further time and information. Patients were also asked if they had discussed end of life wishes with anyone. *Outcome*: Positive outcomes of decision-making. The authors noted the ideal outcome is the decision is consistent with the patient’s goals; however, this was difficult to measure directly so a validated tool for decisional conflict was used as a surrogate. A positive outcome of end of life discussions was measured by the presence of an advanced directive

Ankuda et al. surveyed 1034 patients in the preoperative setting within a single tertiary centre, after they had signed informed consent. Patients were due to undergo a range of procedures, classified in accordance with the 2007 American College of Cardiology/American Heart Association guidelines into minor (e.g. hernia repairs), intermediate (e.g. knee replacement) and major (e.g. open abdominal procedures) for conditions which were categorised as definitely oncological, possible oncological and non-oncological (Fleisher et al. 2007). In the absence of a validated quality metric for preoperative decision-making, the investigators proposed a conceptual framework based on a structure, processes and outcome model (Fig. 1).

Each survey comprised 22 questions directed towards patients comprising basic demographic data, diagnosis and surgical procedure. A further six questions were completed by nurse practitioners to corroborate the patient data alongside additional data such as the presence of an advanced directive.

Specific validated tools were used to measure process and outcome; an adapted control preference scale to ascertain if the decision was patient-led, physician-led or shared and a decision conflict scale to assess the patients’ satisfaction with the decision. In addition, patients were asked if they wanted to speak further about their decisions. As the data generated included both qualitative and quantitative data, mixed methodologies were used in analysis.

A response rate of 77 % was achieved amongst patients, which was substantially more than that required to power the study. Of these 1034 surveys, 533 had the nurse practitioner sections completed. Fifty-six percent (502/895) patients were due to undergo intermediate or major procedures, whilst the remainder were minor in classification. Thirty-six percent of the diagnoses were definitely oncologic in nature according to nurse practitioner surveys, though interestingly only 30 % of patient surveys reported the same classification, so raising the possibility of patients being unaware of an oncological diagnosis.

Fifty-five percent of patients reported decision-making were shared, as opposed to physician-led or patient-led. Those who described their decision-making process as physician-led were deemed to be three times more likely to experience decisional conflict than shared-decision or patient-centred decision-making (*p* < 0.05).

Thirty-four percent of those who completed surveys were considered to have deficits in surgical decision-making, with 13 % defined as critical deficits which imply inadequate informed consent such as lack of knowledge of their diagnosis or procedure. The remainder of deficits were identified as those who had decisional conflict regarding surgery and what mattered to them as an individual or felt they had inadequate support and advice to make a decision. Furthermore, the study identified as significant number of patients reporting they would benefit from further discussion regarding surgery (212/844). Comparable trends were seen in discussions surrounding advanced care plans, with 30 % patients reporting they would like to speak further about their advanced care plans and a similar percentage reporting no previous discussions regarding their end of life wishes with anyone.

Decisional conflict and wanting to speak more about the surgery were both associated with higher preoperative anxiety levels (*p* < 0.05 %), with socioeconomic factors such as language and education level identified as risk factors for poor decision-making.

The authors concluded that current preoperative processes do not consistently provide adequate information and deliberation support for shared decision-making and advanced care planning. They reported more worryingly that minimum standards for informed consent were not met which highlights ethical considerations regarding the appropriateness of surgery in this cohort. The authors suggested that engaging patients in surgical decision-making, through focused interventions, would support high quality care and ultimately patient-centred care.

## Conclusion

To our knowledge, this is the first large scale study investigating preoperative SDM using a Donabedian model. Measuring quality of shared decision-making processes is a challenge; however, this conceptual model may provide a reliable metric for the perioperative setting.

Whilst accepting the limitations of a single centre and survey-based study, the deficits in perioperative decision-making are hard to ignore and should be addressed with urgency if we are to deliver a true model of patient-centred care. We feel these deficits can be broadly addressed through patient and professional engagement.

Joseph-Williams et al. undertook a recent systematic review of patient perceptions concluding patients perceive a ‘power-imbalance’ in healthcare consultations (Jospeh-Williams et al. 2014). There appeared a reticence by patients to fulfil the role of autonomous individuals through factors such as feeling their knowledge of what is best is inferior to clinicians’ and non-compliance with clinicians’ choices will result in worse healthcare. Interventions to first prepare a patient to speak of their wishes and preferences, and then enable and support a deliberation process, show early promise: examples include ‘Ask 3 questions’ or the ‘Talk Model’ (Shepherd et al. 2011; Elwyn et al. 2012, 2013). An innovative manner to engage with patients is the concept of ‘flipped healthcare’; changing the conversation from ‘what’s the matter with you?’ to ‘what matters to you?’ promoting health rather than focusing on disease (Bisognano and Schummers 2014).

It is challenging to engage patients in the process of SDM until professional engagement is achieved: that is, professionals must want and have the resources available to deliver SDM. Shared decision-making involves the timely delivery of accurate, evidence-based and objective information to a patient and providing subsequent deliberation support to process this information. Clinicians may feel they ‘do this already’; however, this study suggests this perception is not played out in reality (Coulter and Collins 2011; Appleby et al. 2011). Perceived time and financial constraints may deter professionals, yet this has been refuted and indeed more time may be spent supporting patients through decisional conflict (Coulter and Collins 2011; Lee and Emanuel 2013).

A significant barrier to the implementation of SDM within perioperative care is lack of professional education and training. Core competencies in shared decision-making are yet to be established (Legare et al. 2013), and currently, SDM does not feature in mandatory perioperative medicine education and training. Raising professional awareness, through education, may support improvement science interventions.

The population presenting for surgery is changing, older with increasing chronic multi-morbidity. This necessitates a change in focus from single healthcare episodes to viewing the bigger picture on the horizon. Surgical interventions should be placed in the context of patients’ beliefs, preferences and wishes in the long term, beyond their hospital stay. Placing the patient at the centre of decision-making is imperative to ethical perioperative practice.

## Abbreviations

ACP, advance care plan; SDM, shared decision-making
